# Impact of the dimple indentation depth and location for passive flow control in Blended Wing Body airframe at low and high subsonic speeds

**DOI:** 10.1371/journal.pone.0325778

**Published:** 2025-07-03

**Authors:** Haris Ali, Mohammad Rasidi Rasani, Zambri Harun

**Affiliations:** 1 Department of Mechanical and Manufacturing Engineering, Faculty of Engineering and Built Environment, Universiti Kebangsaan Malaysia (UKM), Bangi, Selangor, Malaysia; Donghua University, CHINA

## Abstract

This research investigates the role of dimples in enhancing the aerodynamic characteristics of a Blended-Wing-Body (BWB) airframe. Numerical simulations, grounded in Computational Fluid Dynamics (CFD), were utilized to model turbulent airflow and assess the aerodynamic forces acting on the wing structure. The *k-ω* Shear-Stress Transport (SST) turbulence model was applied to effectively solve the governing equations. The impact of four dimple indentation depths (*d/D*_*d*_ = 0.025, 0.05, 0.075, and 0.1) at six specific locations on either the suction or pressure sides of the BWB wing surface was investigated. Simulations were performed at *Mach* 0.15 and *Mach* 0.6, treating the flow as incompressible and compressible, respectively, to capture variations in aerodynamic behavior. The evaluation involved analyzing the drag coefficient (*C*_*D*_), lift coefficient (*C*_*L*_), and lift-to-drag (*L/D*) ratio. The results reveal that, under optimal conditions, a dimpled BWB surface can achieve a reduction in *C*_*D*_ by as much as 4.09% relative to a non-modified surface, without negatively impacting lift. This improvement is primarily due to the dimples’ capacity to maintain attached flow and postpone flow separation. Implementing dimples on the BWB wing surface as a passive flow control method has proven effective in enhancing the aerodynamic efficiency of lifting surfaces.

## 1. Introduction

A significant endeavor among aircraft designers has consistently been directed towards enhancing the aerodynamic efficiency and performance of aerial vehicles through the exploration of innovative configurations and techniques for flow control. In the realm of manned aviation, the configurations have remained relatively stagnant over the past five decades [1], primarily due to the perceived elevated business risk associated with the adoption of new and unconventional configurations [2]. However, in the 21^st^ century, the tailless novel configurations such as the Blended-Wing-Body (BWB) and flying-wing configurations have significantly revolutionized the aviation industry [3].

Liebeck first introduced the concept of the BWB for high-speed subsonic commercial aircraft [4]. Subsequent to this seminal proposal, numerous researchers have explored the potential of this configuration for various applications, including commercial airliners [5–7], Unmanned Air Vehicle (UAV) utilization [8–10], and cargo transport [11]. The integrated design of the BWB, combining high-lift wings with a wide airfoil-shaped body, has garnered attention in numerous investigations for its superior aerodynamic properties and decreased fuel or power consumption. This advantage arises from its reduced wetted area, lower structural weight, and the lift-generating contribution of the airfoil-shaped fuselage, all of which enhance the overall aerodynamic efficiency of the BWB configuration [12]. Several studies have highlighted an enhancement of up to 20% in the *L/D* ratio of BWB configurations, predominantly attributed to the absence of empennage [13,14].

However, despite advancements, the imperative to enhance its aerodynamic efficiency, particularly in drag reduction and improved *L/D* ratio, persists, necessitating innovative solutions for improved efficiency and sustainability in modern aviation. For civil or commercial transport aircraft, regardless of size, viscous or skin friction drag typically constitutes approximately 40–50% of the total drag during cruise conditions. Consequently, even minor reductions in drag yield substantial benefits [15]. Overall, the aerodynamic efficiency of BWB airframes can be optimized through the application of surface flow control techniques, which are effective in reducing skin friction drag [16].

These approaches can be divided into active and passive ways. Active control techniques are preferred because they can respond to a wide range of operating situations. However, these solutions need additional financial investments in energy supplies, control devices, and air systems, which limits their usefulness. Conversely, passive flow control strategies involve altering the geometric shape of the body or incorporating additional components. Passive strategies are regarded as more practical than their active counterparts due to their simplicity and cost-effectiveness. Therefore, passive control approaches are preferred to improve the aerodynamic efficiency of lifting surfaces. These methods influence stall phenomena and delay flow separation without relying on external energy sources [17].

One of the most widely used passive flow control techniques involves installing winglets at the wingtips, primarily to mitigate induced drag resulting from wingtip vortex downwash [18–22]. Another widely used passive method is the implementation of riblets [23–26]. Since NASA’s development of riblets in the 1980s, extensive research has been conducted on their aviation applications. Riblets, characterized by streamwise-aligned microgrooves, have proven effective in reducing friction drag. They come in various cross-sectional shapes, with the triangular profile being the most common, although an extremely sharp tip is a crucial feature. Although recent studies [27,28] indicate a promising potential for riblets in aeronautics, their integration into commercial transport aircraft remains limited. This restriction is largely due to minimal cost benefits and substantial production and maintenance challenges associated with the small size of riblets and the requirement to maintain sharp tip profiles [29,30].

An emerging option that bypasses the complexity of riblets has been developed, characterized by simplified manufacturing processes and the absence of intricate details. This approach involves embedding a pattern of small dimples onto the surface. Dimples, denoting small concavities impressed upon a surface, have been subject to thorough investigation in previous studies owing to their ability to augment surface heat transfer [31]. The use of dimples on bluff bodies, such as golf balls, is well-recognized for its impact on the turbulent boundary layer and flow separation [32]. This technique is also being investigated in the field of sports car racing [33].

Dimples, serving as a type of surface roughness, facilitate the development of a turbulent boundary layer, thereby delaying flow separation, diminishing wake, and reducing form drag. Incorporating dimples into streamlined objects like airfoils at varying angles of attack could potentially minimize wake size and postpone flow separation [34]. Asai et al. [35] conducted wind tunnel experiments on volleyballs featuring dimpled and honeycomb surface textures. The results indicated a slight reduction in the critical Reynolds number for the modified balls, with honeycomb-textured balls displaying a higher drag coefficient than standard models, while dimpled balls exhibited greater variability in flight orientation. Similarly, Joseph et al. [36] performed numerical simulations to analyze the effect of square dimples on the suction side of a NACA0012 airfoil at varying angles of attack, concluding that dimples have the potential to delay the onset of stall.

Tay et al. [37] conducted an experimental investigation into the effects of circular axisymmetric and teardrop-shaped dimples on drag reduction in turbulent channel flow, with Reynolds numbers spanning from 5,000–50,000. The study found that the greatest drag reduction occurred when the sharp tip of the dimples was oriented upstream rather than downstream. Additionally, tear-drop-shaped dimples demonstrated superior drag reduction compared to circular axisymmetric dimples under comparable conditions.

Azlan et al. [38] explored the impact of dimples on the aerodynamic characteristics of the horizontal axis wind turbine (HAWT) through CFD. Dimpled surfaces on the turbine blade’s suction side were found to increase output torque by up to 8.41%. The findings suggest that incorporating dimples can effectively improve the efficiency of wind turbines. In another similar study by Sedighi et al. [39], they analyzed the effects of dimple radius and location on HAWT performance through numerical simulations. The study demonstrated a remarkable 16.08% improvement in torque, thereby highlighting the potential effectiveness of this passive modification in optimizing wind turbine performance.

Building on both experimental and computational techniques, Zhang et al. [40] explored drag reduction in marine vessels. Their findings revealed that excessively large dimples can induce counterclockwise swirls downstream, implying a potential drawback to simply increasing air volume. However, for scenarios without air injection, strategically designed dimple geometries were found to be capable of achieving significant drag reduction, up to 40.38%. Wang et al. [41] utilized computational modeling to achieve drag reduction on a vehicle body by implementing a dimpled, rough surface. This approach altered *C*_*D*_ by minimizing wake vortices and mitigating turbulent kinetic energy. Through numerical analysis, Chear and Dol [42] examined how altering the ratio of dimple depth to diameter influenced the drag coefficient in a car model. Their findings showed that all examined ratios contributed to drag reduction, with the maximum decrease in the *C*_*D*_ reaching 1.95%.

Therefore, over the past two decades, several research groups have dedicated their endeavors to comprehending the issue of drag reduction through dimples. The multitude of dimple design variables awaiting examination presents a formidable challenge, particularly in the absence of a guiding theory, hypothesis, or established scaling argument to direct exploration within this extensive parameter space. Moreover, to date, there has been no investigation into the influence of dimples as a passive flow control method on the aerodynamic performance and flow physics of BWB aircraft. Motivated by this potential, the present study conducts a numerical analysis to assess the effects of surface dimples on the aerodynamic performance of a BWB airframe. The influence of various dimple indentation depths and their positions on the suction and pressure surfaces of BWB is investigated. The dimples are arranged at selected positions along the chord, with a fixed spacing between them. To analyze the airflow, simulations were conducted at *Mach* 0.15 and *Mach* 0.6, treating the flow as incompressible and compressible, respectively. An incompressible Reynolds-Averaged Navier-Stokes (RANS) solver coupled with the *k-ω* SST turbulence model was employed for low-speed cases, while compressibility effects were accounted for at higher speeds. The influence of dimples on the aerodynamic performance of the BWB airframe was assessed by analyzing parameters such as the *C*_*D*_, *C*_*L*_, drag polar and *L/D* ratio. The findings suggest that well-designed dimples hold potential for reducing *C*_*D*_ and improving the *L/D* ratio.

## 2. Problem description

This study utilizes a CFD approach to evaluate the aerodynamic performance of a dimpled BWB airframe. Advancements in computing power have made CFD a popular tool across engineering disciplines. Its strength lies in solving the complex Navier-Stokes equations that govern fluid flow based on the principles of mass, energy, and momentum conservation. CFD employs various turbulence models to account for real-world flow behaviour under different conditions. Additionally, CFD allows for the generation and analysis of a wide range of output results during post-processing. This includes, but is not limited to, visualizing flow streamlines, as well as contours of pressure, temperature, and velocity, along with flow animations and other detailed representations of the solution. These informative visualizations are instrumental in explaining the results and providing deeper insights into intricate flow processes.

### 2.1. Baseline BWB configuration

**Fig 1:**
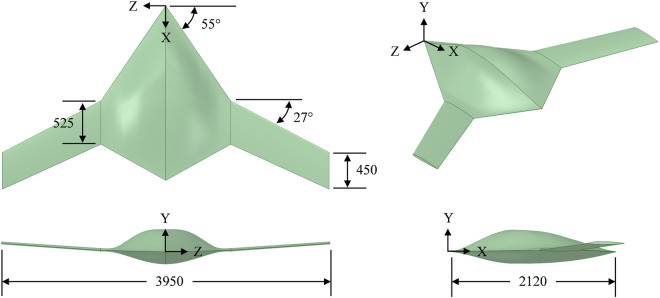
External layout of BWB airframe, all dimensions are in millimeters (mm).

The baseline configuration employed in this study adopts an optimized fixed-wing BWB airframe geometry [43]. Airfoil profiles were extracted at various spanwise locations, extending from the central body to the outer wing, inclusive of the wing tip. These profiles functioned as the foundational elements for generating all geometries investigated in this study. The root chord, *c*_*r*_, at the center of the body measures 2.12m, the tip chord, *c*_*t*_, at the outermost section of the wing is 0.45m, and the kink chord, *c*_*k*_, at the intersection of the body and wing is 0.525m. The body exhibits a sweep angle, ∧_*b*_, of 55°, while the wing showcases a sweep angle, ∧_*w*_, of 27°. Further details regarding the design procedure, tools, and layout can be found in [43]. The external layout of the reference BWB airframe, presented in Fig 1, serves as the benchmark configuration for the integration of dimples. The fundamental geometric details pertinent to the current investigation are presented in Table 1.

**Table 1:** Baseline BWB airframe specifications.

Parameter	Definition/value
Body sweep angle, ∧_*b*_	55°
Wig sweep angle, ∧_*w*_	27°
Body taper ratio, *λ*_*b*_	0.25
Wing taper ratio, *λ*_*w*_	0.85
Root chord, *c*_*r*_	2.12m
Kink chord, *c*_*k*_	0.525m
Tip chord, *c*_*t*_	0.45m
Mean aerodyamic chord, *MAC*	0.91m
Reference span, *b*_*ref*_	3.95m
Reference area, *S*_*ref*_	3.50m^2^

### 2.2. Dimple design characterization

**Fig 2:**
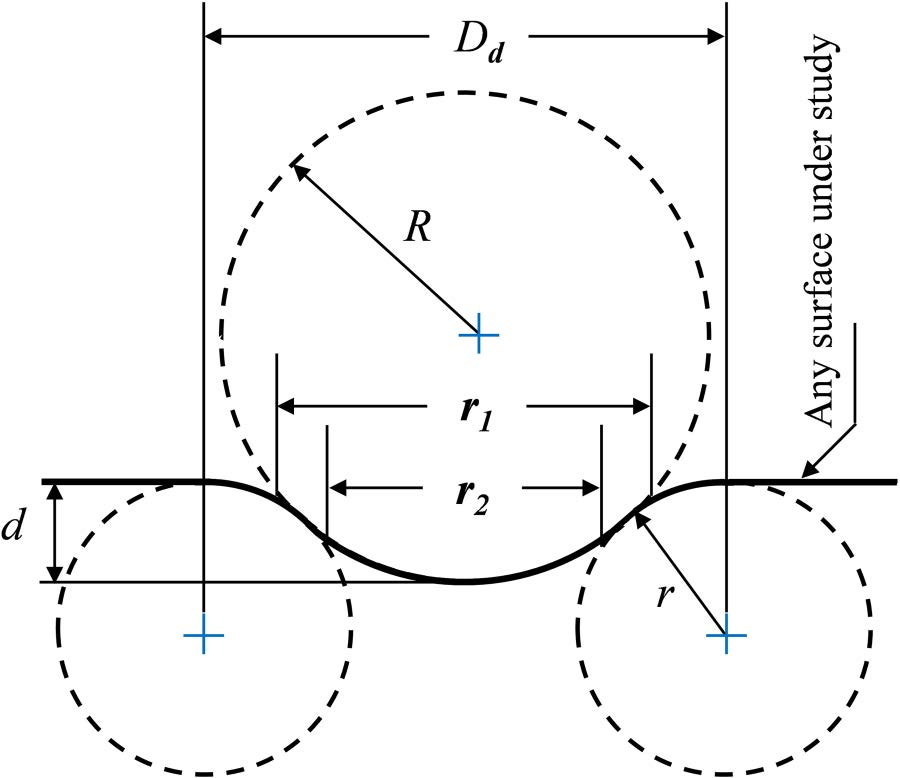
Schematic of spherical dimple geometric parameters (depth is exaggerated for illustration purpose).

A planar solid surface modified with dimples is characterized by various geometric parameters. Emphasizing the significance of a precisely defined parametric geometry is crucial in the pursuit of achieving optimal dimple performance. Originally conceived as spherical recesses with circular footprints on the surface, a particular type of circular dimple, as introduced by Chen et al. [44], has gained popularity due to its parametric nature and is utilized in our present investigation. This dimple design arises from the combination of a spherical indentation and a torus, intersecting tangentially in a uniform manner to eliminate sharp edges. The cross-sectional view of this axially symmetrical dimple is shown in Fig 2.

The geometric attributes of the particular dimple are defined by four key variables, which are interrelated through an analytical relationship, consequently constraining the system to three degrees of freedom. The following analytical formula establishes the relationship among these dimple parameters [17]:


$$D_{d}=2\sqrt{d(2R+2r-d)}$$ (1)

here,

*D*_*d*_ = diameter of the circular dimple.

*d* = indentation depth of the spherical ball.

*r* = curvature radius at the intersection of wall and spherical ball.

*R* = curvature radius of the spherical ball, $R=\;\frac{d}{2}+\;\frac{r_{1}^{2}}{8d}$

*r*_*2*_ = internal region radius, $\frac{R}{R+r}D_{d}$

### 2.3. BWB configurations with dimples

**Fig 3:**
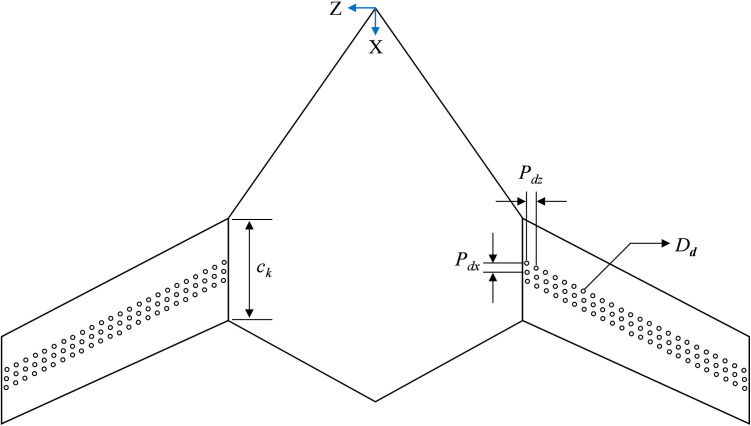
2-D top view of BWB airframe integrated with dimples (schematic shown for *X*_*ss*_*/c*_*k*_ = 0.5).

This study, as outlined earlier, conducts a numerical investigation into how surface dimples affect the aerodynamic performance of a BWB airframe. The influence of various dimple depths and locations on the suction and pressure surfaces of BWB is investigated. The dimples integrated on the BWB surface are limited to the patterns of spherical dimples with a dimple diameter, *D*_*d*_, of 0.025*c*_*k*_. All lengths are made non-dimensional with respect to *D*_*d*_. Several authors [45–47] report that shallower dimples with dimple depth-to-diameter ratio, *d/D*_*d*_, less than 10% should be considered for drag reduction purposes. Based on this literature review, shallow dimples with *d/D*_*d*_ of 2.5, 5, 7.5 and 10% are considered for investigation in this study. All dimples have a rounding radius of *r*/*D*_*d*_* *= 0.5, resulting in curvature radii of *R*/*D*_*d*_* *= 4.51, 2.03, 1.20, and 0.8 for the studied dimple depths, respectively. The surface of BWB wing is modified by placing three rows of spherical dimples on the suction and pressure sides either at 15%, 50% or 85% of the chord length starting from the leading edge (LE) to the trailing edge (TE). This deliberate arrangement aims to identify optimal locations for dimple placement, thereby enhancing aerodynamic effectiveness. The dimples in this study have a center-to-center spanwise dimple spacing, *P*_*dz*_, and streamwise dimple spacing, *P*_*dx*_, of 1.5*D*_*d*_, where *x* is defined in the direction of the free stream velocity, as shown in Fig 3. The simulations are performed at low and high subsonic velocities to analyze the dimple’s performance over the range of angle of attacks to cover all the flight regimes. All simulations are conducted at sea level conditions, assuming fully turbulent flow. Reynolds numbers $\left(Re=\frac{\rho VL}{\mu }\right)$ for both speeds are calculated using *MAC* as the characteristic length. Tables 2 and 3 provide a summary of the geometric characteristics of the dimple configurations studied, along with the flow parameters used for analysis.

**Table 2:** Geometric parameters of dimples integrated on BWB surface.

Parameter	Definition/value
*Parameters which are kept constant*	
Dimple shape	Spherical
Dimple diamater, *D*_*d*_	0.025*c*_*k*_
Spanwise dimple spacing, *P*_*dz*_	1.5*D*_*d*_
Streamwise dimple spacing, *P*_*dx*_	1.5*D*_*d*_
Dimple Layout	Staggered
Edge curvature radius, *r*	0.5*D*_*d*_
*Parameters which are varied for analysis*	
Dimple depth, *d*	0.025, 0.05, 0.075 & 0.1*D*_*d*_
Dimple placement location	
- Suction surface, *X*_*ss*_	0.15, 0.5 & 0.85*c*_*k*_
- Pressure surface, *X*_*ps*_	0.15, 0.5 & 0.85*c*_*k*_

**Table 3:** Input parameters of flow physics.

Parameter	Definition/value
Free stream velocity, *V*_*∞*_	
• Low subsonic	*Mach* 0.15
• High subsonic	*Mach* 0.6
Angle of attack, *α*	−2° to 6° (Δα = 2°)
Altitude	Sea level
Reynolds No., *Re*	
• Low subsonic	3.2 × 10^6^
• High subsonic	1.3 × 10^7^

## 3. Governing equations and boundary conditions

To simulate the flow around the BWB, a steady-state approach was employed with consideration of both incompressible and compressible regimes, depending on the flow conditions. Specifically, for low-speed conditions (*Mach* 0.15), the flow was treated as incompressible, and a pressure-based RANS solver was used. For high-speed conditions (*Mach* 0.6), compressibility effects were accounted for using a density-based solver. In both cases, the *k-ω* SST turbulence model was adopted to ensure accurate prediction of near-wall behavior and free-stream dynamics. The fundamental governing equations for the incompressible case include the continuity and momentum equations, expressed as follows [48]:


$$\frac{\partial {\overset{―}{u}}_{i}}{\partial x_{i}}=0$$ (2)

$$\frac{\partial \left(\rho {\overset{―}{u}}_{i}\right)}{\partial t}+\frac{\partial \left(\rho \overset{―}{u_{i}u_{j}}\right)}{\partial x_{j}}=-\frac{\partial \overset{―}{p}}{\partial x_{j}}+\frac{\partial }{\partial x_{j}}\left(\mu \frac{\partial {\overset{―}{u}}_{i}}{\partial x_{j}}-\rho \overset{―}{u_{i}^{\prime }u_{j}^{\prime }}\right)$$ (3)

here,

*ū* and *u’* = average and instantaneous components of velocity, respectively.

*p* = pressure.

*ρ* = fluid density.

*x*_*i*_ and *x*_*j*_ = cartesian coordinates.

*t* = time.

*µ* = fluid dynamic viscosity.

For the high speed at Mach 0.6, where compressibility effects become significant, a density-based solver was used in ANSYS Fluent to solve the full Navier-Stokes equations, including conservation of mass, momentum, and energy. The fluid was assumed to behave as a perfect gas, and the ideal gas law was applied as the equation of state. The compressible governing equations are expressed as follows:


$$\frac{\partial \rho }{\partial t}+\frac{\partial \left({\rho u}_{j}\right)}{\partial x_{j}}=0$$ (4)

$$\frac{\partial \left(\rho u_{i}\right)}{\partial t}+\frac{\partial \left(\rho u_{i}u_{j}\right)}{\partial x_{j}}=\;-\frac{\partial p}{\partial x_{i}}+\frac{\partial \tau_{ij}}{\partial x_{j}}$$ (5)

where the stress tensor *τ*_*ij*_ is given by:


$$\tau_{ij}=\;\mu \left(\frac{\partial u_{i}}{\partial x_{j}}+\frac{\partial u_{j}}{\partial x_{i}}-{\frac{2}{3}\delta }_{ij}\frac{\partial u_{k}}{\partial x_{k}}\right)$$ (6)

$$\frac{\partial (\rho E)}{\partial t}+\frac{\partial \left[u_{j(\rho E\;+\;p)}\right]}{\partial x_{j}}=\frac{\partial }{\partial x_{j}}\left(k\frac{\partial T}{\partial x_{j}}+\;u_{i}\tau_{ij}\right)$$ (7)

where *E* is the total energy per unit mass, defined as:


$$E\;=\;e\;+\;0.5\left(u_{1}^{2}+\;u_{2}^{2}+\;u_{3}^{2}\right)$$ (8)

with *e* being the internal energy, and *k* the thermal conductivity.


p = ρRT (9)

The *k-ω* SST turbulence model is well-regarded for its ability to merge the strengths of the *k-ω* model within the boundary layer’s inner regions and the *k-ε* model in the free stream. The full equation for this model is provided below [49]:


$$\frac{\partial (\rho k)}{\partial t}+\frac{\partial \left(pku_{i}\right)}{\partial x_{i}}=\frac{\partial }{\partial x_{j}}\left(\Gamma_{k}\frac{\partial k}{\partial x_{j}}\right)+G_{k}-Y_{k}+S_{k}$$ (10)

$$\frac{\partial }{\partial t}(\rho \omega )+\frac{\partial }{\partial x_{i}}\left(\rho \omega u_{i}\right)=\frac{\partial }{\partial x_{j}}\left(\Gamma_{\omega }\frac{\partial \omega }{\partial x_{j}}\right)+G_{\omega }-Y_{\omega }+D_{\omega }+S_{\omega }$$ (11)

here,

*k* = turbulence kinetic energy.

*ω* = specific heat dissipation rate.

*G*_*k*_ = generation of turbulence kinetic energy due to average velocity gradients.

*G*_*ω*_ = generation of *ω*.

*Г*_*k*_ and *Г*_*ω*_ = effective diffusivity of *k* and *ω*, respectively.

*Y*_*k*_ and *Y*_*ω*_ = dissipation of *k* and *ω* due to turbulence.

*D*_*ω*_ = cross-diffusion term.

*S*_*k*_ and *S*_*ω*_ = user-defined source terms.

**Fig 4:**
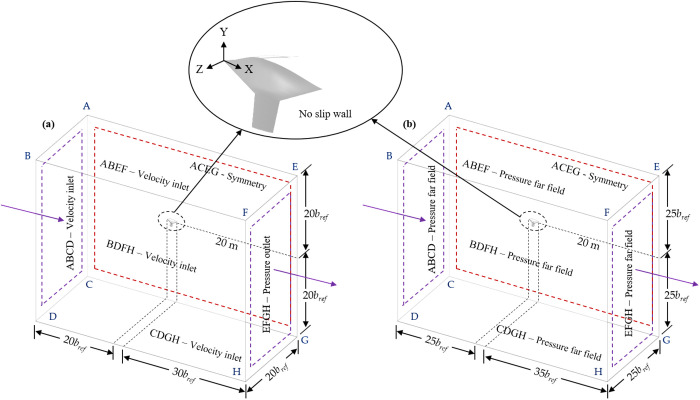
Demonstration of boundary conditions and computational domain; (a) Low-subsonic flow; (b) High-subsonic flow.

This study is carried out numerically using ANSYS Fluent within a fixed (inertial) reference frame (FRF). The FRF approach assumes that the coordinate system is stationary and does not move along with the flow, making it ideal for analyzing external aerodynamic flows, such as those around an aircraft or wing. Fig 4 illustrates the boundary conditions and the geometry contained within the defined domain. Two distinct sets of boundary conditions are employed for low subsonic and high subsonic velocities to accurately resolve the flow field. In this study, the BWB airframe, modified with spherical dimples, is positioned within a fixed rectangular enclosure to simulate the wind field. For external flow simulations, an enclosure size approximately 15–25 times the *c*_*r*_ upstream and 25–40 times *c*_*r*_ downstream is deemed suitable.

For the low-subsonic flow scenario, the simulations assume steady-state and incompressible airflow. The AOA is applied by vectoring the freestream velocity direction at the upstream boundary. To ensure uniform inflow and suppress boundary-induced disturbances, velocity inlet conditions are applied on the upstream (front), top, bottom, and left (spanwise) faces of the enclosure. All inlets use the same velocity magnitude (50 m/s) and vector orientation, ensuring consistent freestream conditions aligned with the BWB chord line across the domain. The right face of the enclosure is modeled as a symmetry boundary, since only half of the BWB airframe is modeled to reduce computational cost. The rear face (downstream) is defined as a pressure outlet, maintaining gauge pressure at zero to allow subsonic flow to exit naturally. All solid walls, including the BWB and dimple surfaces, are assigned no-slip wall conditions.

For the high-subsonic flow, compressibility effects are accounted for by treating air as an ideal gas and employing a density-based solver. The same geometric configuration is used, but a pressure far-field boundary condition is applied to all outer boundaries of the enclosure. This ensures proper handling of the weak compressibility effects that arise beyond *Mach* 0.3. The pressure far-field setup allows the solver to capture variations in density, pressure, and temperature, enabling a realistic representation of the aerodynamic behavior at *Mach* 0.6. As with the low-speed case, a symmetry condition is applied to the mid-span plane, and the no-slip wall condition is used for the BWB surface.

To define turbulence properties at all inlet and outer boundaries, a turbulence intensity of 5% and turbulent viscosity ratio of 10 were applied. These values represent the default recommendations in ANSYS Fluent for external aerodynamic simulations, and were retained to ensure solver stability and minimize arbitrary user intervention. Such parameters are widely adopted in the literature, particularly when case-specific experimental turbulence data are unavailable. The solver employs a relative velocity approach, with pressure-velocity coupling handled via the COUPLED scheme. A ‘second-order upwind spatial discretization’ method is applied to both pressure and momentum equations, while gradient calculations are performed using a least-squares cell-centered algorithm. The convergence threshold for all residuals is set to 10^−6^.

Two key factors determine an aircraft’s aerodynamic performance: lift and drag. The lift coefficient (*C*_*L*_) represents the lift force (*L*) produced by the wings, which is essential for evaluating the aerodynamic forces acting on the aircraft during flight and ensuring the structural design can endure these forces. Conversely, the drag coefficient (*C*_*D*_) measures the drag force (*D*), which opposes the aircraft’s forward movement. Minimizing drag is crucial for enhancing fuel efficiency, as reduced drag means the engines require less power to maintain a target speed, ultimately decreasing fuel usage. The following equations define these key aerodynamic parameters [50]:


$$C_{L}=\frac{L}{0.5\rho V_{\infty }^{2}S_{ref}}$$ (12)

$$C_{D}=\frac{D}{0.5\rho V_{\infty }^{2}S_{ref}}$$ (13)

here,

*V*_*∞*_ = free stream velocity.

*S*_*ref*_ = reference area of the BWB.

## 4. Meshing and sensitivity analysis

This research utilized a blend of structured and unstructured meshing methods. A structured mesh was applied near the BWB surface to capture the flow details and abrupt changes (sharp gradients) within the boundary layer more effectively, despite resulting in a localized increase in element count. For the larger computational domain, an unstructured mesh was used, providing greater flexibility in handling the complex 3D flow configurations around the BWB. To ensure accurate boundary layer representation for turbulent flow simulations using the *k-ω* SST turbulence model, the y+ parameter is kept below 5 [51]. This approach maintains sufficient node density near the walls to resolve the viscous sub-layer accurately. To meet this criterion, twenty prism layers are added close to the walls, with specific values for the initial layer height and growth rate tailored for low and high subsonic free stream velocities, as outlined in Table 4. These mesh settings are uniformly applied across all dimpled BWB configurations at both Mach numbers. Calculations for the initial layer height, necessary to achieve the targeted y+ value, are derived using flat plate flow approximations, as shown in equations (14) and (15).

**Table 4:** Estimation of inflation layers.

Parameter	Low-subsonic (*Mach* 0.15)	High-subsonic (*Mach* 0.6)
Target y+ value	≤1	≤1
First layer height	1.5 × 10^−5^ m	4.2 × 10^−6^ m
Growth rate	1.35	1.44
Boundary layer thickness	1.74 × 10^−2^ m	1.32 × 10^−2^ m


$$\Delta y=\frac{y^{+}\mu }{\rho u^{*}}$$ (14)

$$u^{*}=\sqrt{\frac{\tau_{\omega }}{\rho }}$$ (15)

here,

Δy = height of the first layer.

y+ = target y+ value.

*μ* = dynamic viscosity.

*ρ* = air density.

u* = friction velocity.

*τ*_*ω*_ = wall shear stress.

**Fig 5:**
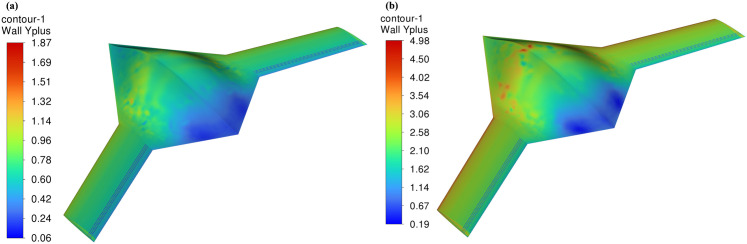
y+ contours shown for *X*_*ss*_*/c*_*k*_ = 0.85; *d/D*_*d*_ = 0.05; (a) *Mach* 0.15; (b) *Mach* 0.6.

The y+ contours for both flow conditions are shown in Fig 5. For the low-subsonic case, Fig 5a illustrates that y+ values remain consistently below 2 across the entire surface of the BWB, which is ideal for resolving the viscous sublayer directly. This level of resolution aligns with the low *Re* formulation of the *k–ω* SST turbulence model, which does not rely on wall functions and benefits from fine near-wall mesh.

For the high-subsonic case, Fig 5b indicates a modest increase in y + due to higher flow velocity and wall shear. However, the entire surface still maintains y+ < 5, with the majority of values falling in the range of 1–4. Importantly, the y+ values near the dimpled regions remain close to 1, ensuring that the boundary layer behaviour in these critical zones is accurately captured. The *k–ω* SST model is known for its robustness in this moderate y+ regime, and mesh independence testing further confirmed that aerodynamic performance metrics remained stable with additional refinement. Thus, the mesh was carefully designed to achieve a practical balance between near-wall fidelity and computational feasibility, particularly under compressible flow conditions at *Mach* 0.6.

**Fig 6:**
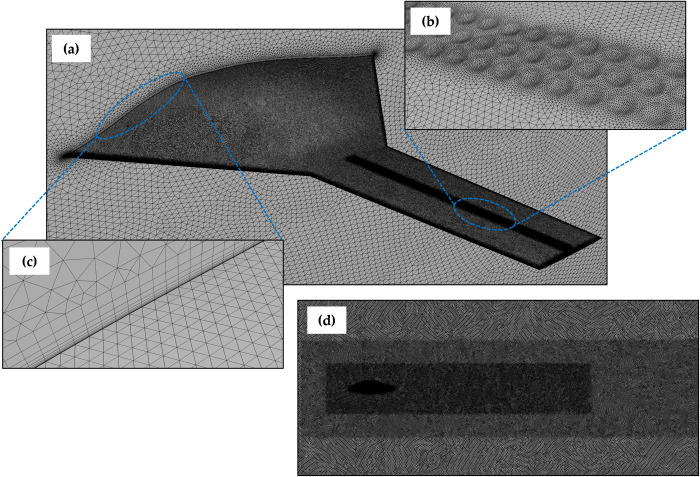
Mesh implementation; (a) around the BWB; (b) on the dimples; (c) inflation layers to capture boundary layer; (d) wake refinement.

Apart from boundary layer thickness, node density was kept high near the LE and TE to accurately capture steep pressure gradients, as well as around the dimples to precisely represent their aerodynamic effects. Additionally, the wake region was refined to accurately capture the flow structures and vortices that form downstream of the BWB wing, ensuring that the wake behavior is well-represented in the simulations. After conducting a thorough mesh independence study, the total mesh element count was set at approximately 8.1 million for *Mach* 0.15 and 12.9 million for *Mach* 0.6. Mesh quality was maintained with an orthogonal quality above 0.15 and skewness below 0.65. Fig 6a and 6b illustrate the application of the computational mesh on the BWB surface, in the domain, and near the dimples, while Fig 6c and 6d provide a visual representation of inflation layers for capturing the boundary layer and the mesh refinement in the wake region, respectively.

To ensure the robustness and reliability of our CFD simulations, a comprehensive grid independence study was conducted to ascertain the influence of grid refinement on the numerical results. In this study, a BWB integrated with dimples of *d/D*_*d*_ = 0.05 on the suction side near the TE was selected for evaluation. The assessment covered both low and high subsonic free stream flow velocities at 0°, 2°, 4°, and 6° angles of attack, using different numbers of mesh elements. The grid independence for both *Mach* numbers was determined by evaluating the convergence of results across different mesh resolutions. The criterion for selection was the comparison of *C*_*D*_ values, and the mesh with minimal deviation was chosen to be representative. For *Mach* 0.15, three different grids were generated, while for *Mach* 0.6, four distinct grids were created. Each set of mesh was refined with consideration for various aspects of the geometry and the flow physics. Refinement in the radial direction was applied to improve the resolution of the flow near the BWB surface, ensuring accurate capture of boundary layer characteristics and flow separation. In the chordwise direction, a denser mesh was used, particularly near the LE and TE, to capture significant pressure gradients. Mesh refinement in the spanwise direction was uniformly applied across the wing to ensure accurate resolution of the flow along the entire span. Special attention was given to the regions surrounding the dimples, where the mesh was significantly refined to accurately represent dimple geometry and capture localized flow effects, including potential flow separation or reattachment phenomena. The detailed specification of these grids is provided in Table 5.

**Table 5:** Details of mesh independence study performed on *X*_*ss*_*/c*_*k*_ = 0.85; *d/D*_*d*_ = 0.05.

Mesh name	No. of mesh elements (×10^6^)	
	(*Mach* 0.15)	(*Mach* 0.6)
Mesh # 1	4.30	4.68
Mesh # 2	8.11	8.75
Mesh # 3	12.24	12.98
Mesh # 4	–	18.17

**Fig 7:**
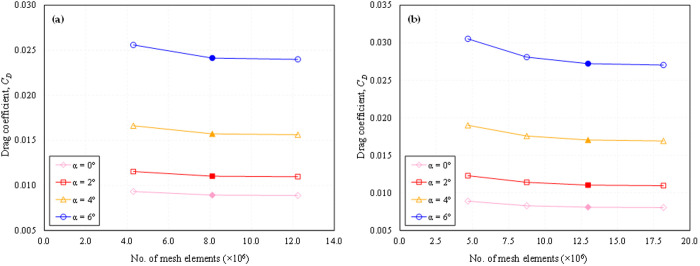
Mesh independence study (drag coefficient, *C*_*D*_) performed on *X*_*ss*_*/c*_*k*_ = 0.85; *d/D*_*d*_ = 0.05; (a) *Mach* 0.15; (b) *Mach* 0.6.

Fig 7 shows that the difference in *C*_*D*_ between mesh 2 and mesh 3 at *Mach* 0.15 is less than 0.6%, and for *Mach* 0.6, the deviation between mesh 3 and mesh 4 is less than 0.7%, observed at the maximum analyzed AOA (6°). Therefore, to optimize computational efficiency and time, mesh 2 was selected for *Mach* 0.15, while mesh 3 was chosen for *Mach* 0.6, each with 8.11 and 12.98 million elements, respectively, for subsequent simulations. The selected grids represent a balanced compromise between computational efficiency and accuracy, providing a solid foundation for subsequent analyses and insights into the aerodynamic behavior of the BWB with dimples.

## 5. Results and discussion

### 5.1. CFD method validation

In addition to the mesh independence study, the reliability of the present investigation was further verified by validating the numerical methodology using the ONERA M6 wing, a widely studied aerodynamic benchmark with extensive experimental data. The simulation results were compared against the surface pressure coefficient (*Cp*) data reported by Schmitt et al. [52]. The ONERA M6 wing geometry was sourced from [53].

The validation was performed at a freestream *Mach* number of 0.84 and an AOA of 3.06°, corresponding to the original wind tunnel test conditions. Based on the mean aerodynamic chord (*MAC* = 0.64607 m), the *Re* number was calculated to be 11.7 × 10⁶. The computational setup employed the same meshing strategy, numerical schemes, and turbulence model (*k–ω* SST) as those used in the dimpled BWB simulations. A near-wall resolution of y+ ≈ 1 was maintained across the wing surface to accurately resolve the boundary layer.

**Fig 8:**
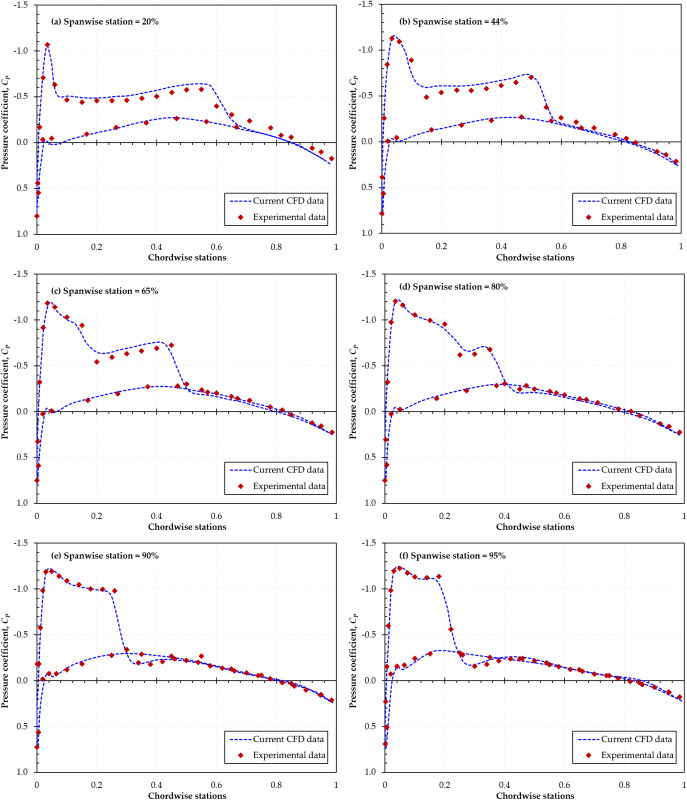
Comparison of experimental [52] and simulated *Cp* distribution at six spanwise locations for the ONERA M6 wing (*Mach* 0.84, AOA = 3.06°).

To assess accuracy, the simulated surface *Cp* distributions were compared with experimental measurements at six spanwise stations. As shown in Fig 8, the numerical predictions exhibit excellent agreement with the experimental data in both trend and magnitude, particularly around the LE and upper surface pressure recovery regions.

These results confirm the robustness of the numerical methodology and the turbulence model adopted in this study. The close correlation with experimental data underscores the validity of the solver setup, thereby reinforcing the credibility of the aerodynamic results presented for the dimpled BWB configurations.

### 5.2. Aerodynamic characteristics of BWB integrated with dimples

Through a numerical analysis, this research explores the effects of incorporating dimples on the wing surfaces of a BWB airframe. Three rows of spherical dimples are integrated to the wing, each featuring specific pitch distance between them and a diameter of *D*_*d*_ = 0.025*c*_*k*_. The study examines the influence of four distinct dimple indentation depths (*d* = 0.025, 0.05, 0.075, and 0.1*D*_*d*_) at six specific locations on either the suction or pressure sides. These locations are situated at 15%, 50%, or 85% of the chord length, extending from the LE to the TE. The simulations are conducted at two distinct speeds, *Mach* 0.15 and *Mach* 0.6, providing a thorough understanding of the impact of dimples on the aerodynamic behavior of the BWB under varying flow conditions.

**Fig 9:**
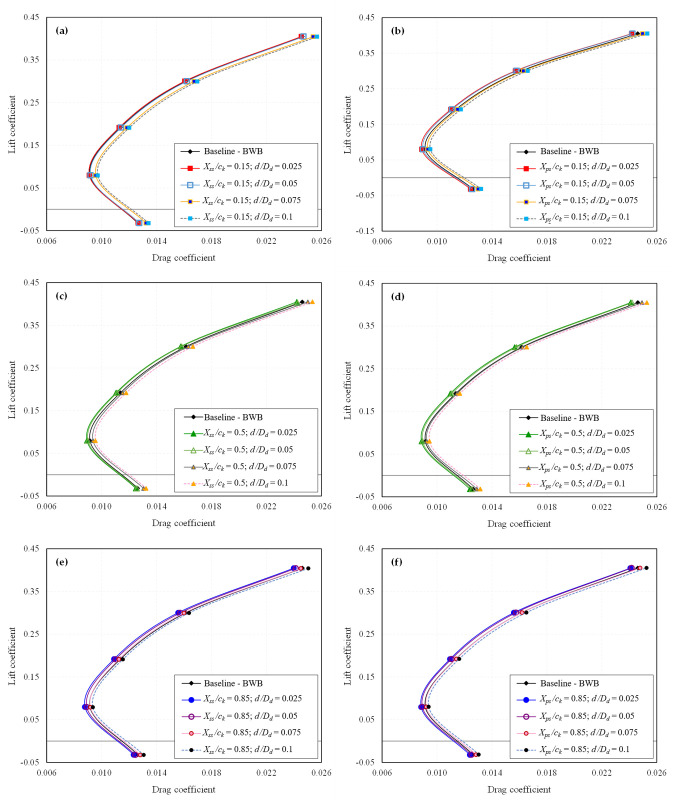
Drag polar of all dimpled BWB configurations at *Mach* 0.15; (a) *X*_*ss*_*/c*_*k*_ = 0.15; (b) *X*_*ps*_*/c*_*k*_ = 0.15; (c) *X*_*ss*_*/c*_*k*_ = 0.5; (d) *X*_*ps*_*/c*_*k*_ = 0.5; (e) *X*_*ss*_*/c*_*k*_ = 0.85; (f) *X*_*ps*_*/c*_*k*_ = 0.85.

**Fig 10:**
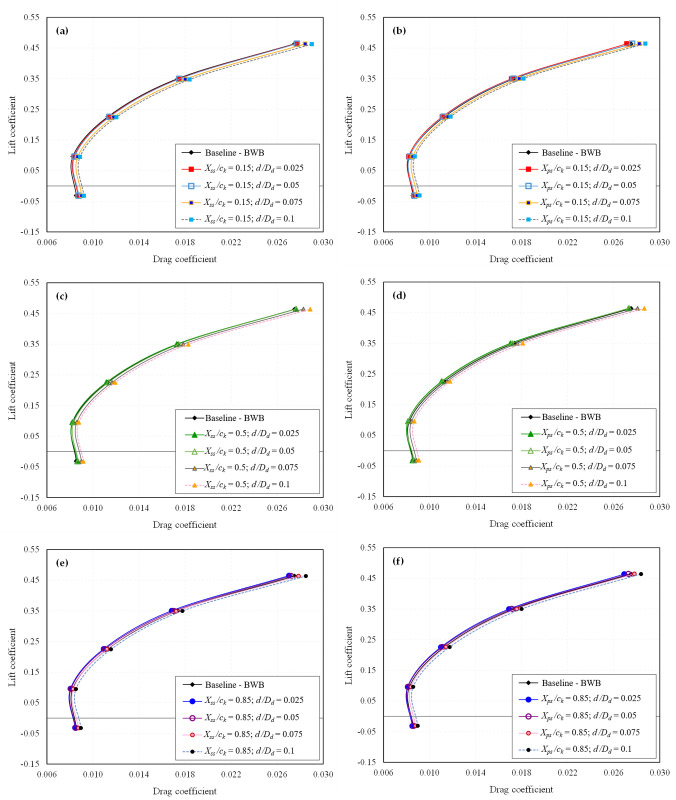
Drag polar of all dimpled BWB configurations at *Mach* 0.6; (a) *X*_*ss*_*/c*_*k*_ = 0.15; (b) *X*_*ps*_*/c*_*k*_ = 0.15; (c) *X*_*ss*_*/c*_*k*_ = 0.5; (d) *X*_*ps*_*/c*_*k*_ = 0.5; (e) *X*_*ss*_*/c*_*k*_ = 0.85; (f) *X*_*ps*_*/c*_*k*_ = 0.85.

**Fig 11:**
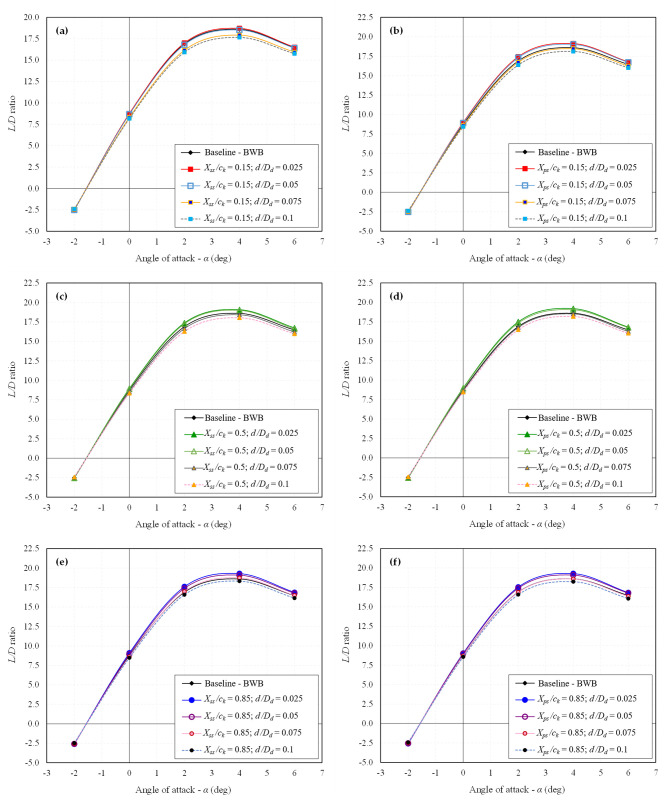
*L/D* ratio of all dimpled BWB configurations at *Mach* 0.15; (a) *X*_*ss*_*/c*_*k*_ = 0.15; (b) *X*_*ps*_*/c*_*k*_ = 0.15; (c) *X*_*ss*_*/c*_*k*_ = 0.5; (d) *X*_*ps*_*/c*_*k*_ = 0.5; (e) *X*_*ss*_*/c*_*k*_ = 0.85; (f) *X*_*ps*_*/c*_*k*_ = 0.85.

**Fig 12:**
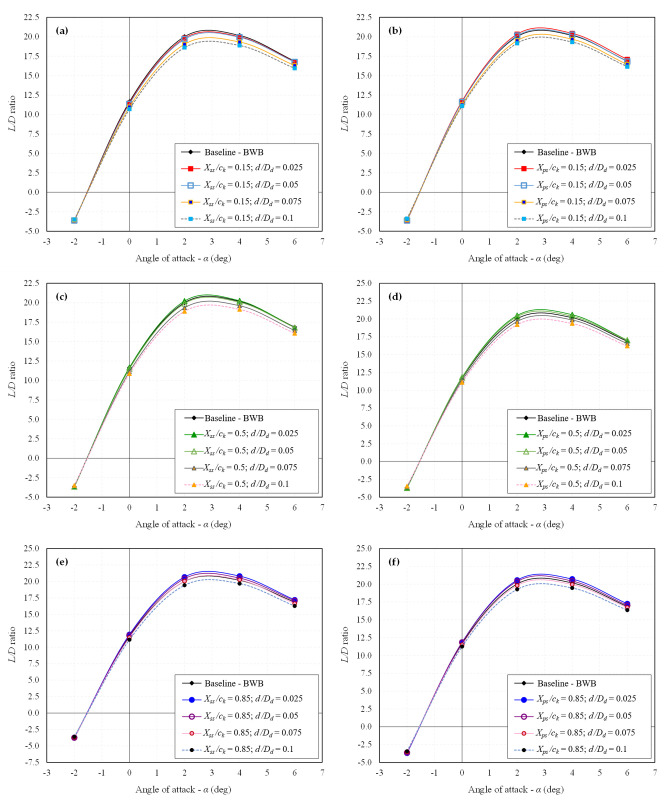
*L/D* ratio of all dimpled BWB configurations at *Mach* 0.6; (a) *X*_*ss*_*/c*_*k*_ = 0.15; (b) *X*_*ps*_*/c*_*k*_ = 0.15; (c) *X*_*ss*_*/c*_*k*_ = 0.5; (d) *X*_*ps*_*/c*_*k*_ = 0.5; (e) *X*_*ss*_*/c*_*k*_ = 0.85; (f) *X*_*ps*_*/c*_*k*_ = 0.85.

The primary stage involves extracting the CFD results for all 24 configurations individually. Essential aerodynamic coefficients such as *C*_*L*_, *C*_*D*_, and *L/D* ratio are computed. The influence of dimples on the aerodynamic characteristics of the BWB airframe is elucidated through drag polar and *L/D* ratio analysis. The drag polar for all the studied dimpled BWB configurations is presented in Figs 9 and 10 for *Mach* 0.15 and *Mach* 0.6, respectively. Similarly, the *L/D* ratio for all the studied dimpled BWB configurations is depicted in Figs 11 and 12 for *Mach 0.15* and *Mach* 0.6, respectively. The results are also compared with those of the baseline BWB configuration (without dimples).

It can be observed that the shallow dimples exhibited the most favorable aerodynamic performance in terms of drag reduction. However, increasing the dimple depth beyond 5% resulted in diminishing efficacy, with depths of 7.5% and 10% actually increasing drag. Similar trends were observed at both Mach numbers, although the efficacy of dimples in terms of drag reduction was reduced at *Mach* 0.6 compared to *Mach* 0.15.

Regarding dimple locations, dimples positioned near the TE on the suction surface exhibited the most significant drag reduction. Conversely, dimples located near the LE did not offer any aerodynamic advantage and, in most instances, led to increased drag. Dimples situated on the pressure side near the TE also demonstrated drag reduction, albeit to a lesser extent.

In contrast, the drag polar graphs reveal that the dimpled configurations show minimal variation in *C*_*L*_ compared to the baseline BWB. Furthermore, as there is an improvement in *C*_*D*_ with no significant change in *C*_*L*_, it results in an enhanced *L/D* ratio, as illustrated in Figs 11 and 12.

### 5.3. Pressure coefficient distribution

**Fig 13:**
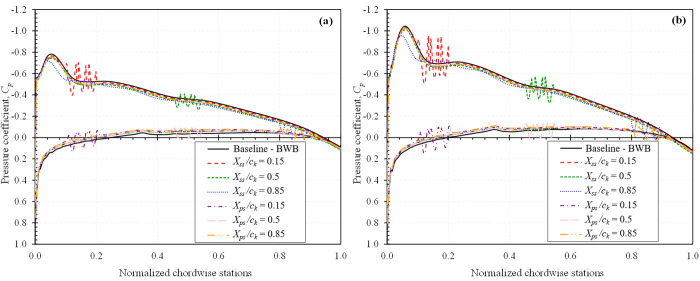
*Cp* distribution of all studied dimple locations for *d/D*_*d*_ = 0.025 at spanwise station *Z/b*_*ref*_ = 1.02; (a) *Mach* 0.15; (b) *Mach* 0.6.

**Fig 14:**
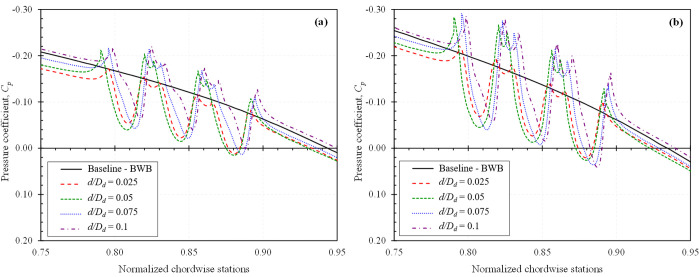
*Cp* distribution of all studied dimple depths for *X*_*ss*_*/c*_*k*_ = 0.85 at spanwise station *Z/b*_*ref*_ = 1.02; (a) *Mach* 0.15; (b) *Mach* 0.6.

To gain a deeper understanding of how dimples influence the aerodynamic performance of a BWB platform, the surface pressure coefficient, *C*_*p*_ distributions were analyzed for configurations that exhibited the highest *L/D* ratio increase, which is at α = 2° for both *Mach* 0.15 and *Mach* 0.6. Fig 13 presents the *C*_*p*_ distributions for all studied dimple locations with a *d/D*_*d*_ = 0.025, while Fig 14 shows the effect of varying indentation depths at the optimal location *X*_*ss*_*/c*_*k*_ = 0.85, both evaluated at a spanwise section *Z/b*_*ref*_ = 1.02.

The plots are rendered with an inverted Y-axis convention: the upper portion of each curve corresponds to the suction surface and the lower portion to the pressure surface. The introduction of dimples generates localized pressure variations that significantly influence the boundary layer flow. As the flow encounters a dimple, it accelerates along the leading face, resulting in a pressure drop. This is typically followed by recirculation within the dimple cavity, which causes a localized pressure rise near the trailing face. This sequence creates a characteristic pressure dip and recovery pattern that is not present on a smooth surface.

These modifications to the *C*_*p*_ distribution suggest enhanced momentum exchange within the boundary layer, promoting flow attachment and delaying separation. In particular, the pressure recovery is smoother and more rapid on the dimpled surfaces, supporting the observed reductions in pressure drag and improvements in aerodynamic efficiency.

### 5.4. Effect of dimple position and indentation depth at low subsonic flow

Investigating the effects of dimples at a low subsonic speed of *Mach* 0.15 provides insights into the aerodynamic performance of the BWB under typical cruising conditions. At this speed, the flow around the aircraft is characterized by relatively lower velocities, resembling scenarios encountered during takeoff, landing, and cruising at subsonic speeds. Analyzing the impact of dimples at *Mach* 0.15 allows us to assess their effectiveness in reducing drag, and improving overall aerodynamic efficiency during these crucial flight phases.

**Fig 15:**
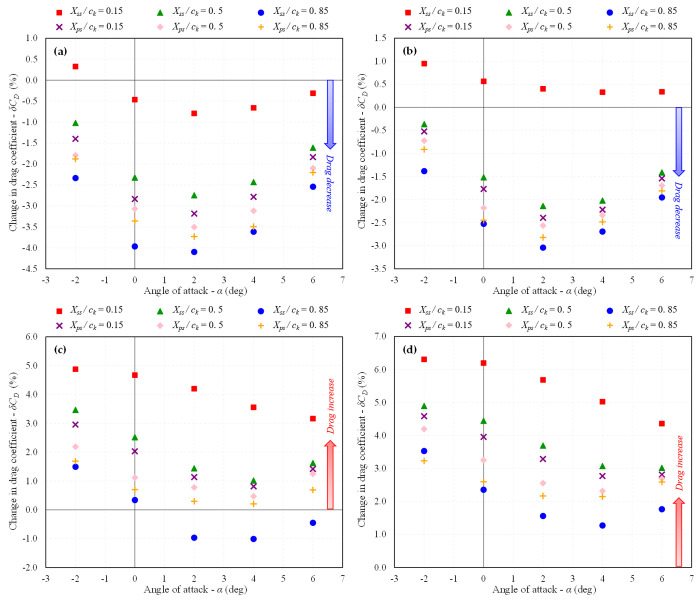
Variation in *C*_*D*_ w.r.t baseline BWB at *Mach* 0.15; (a) *d/D*_*d*_ = 0.025; (b) 0.05; (c) 0.075; (d) 0.1.

**Fig 16:**
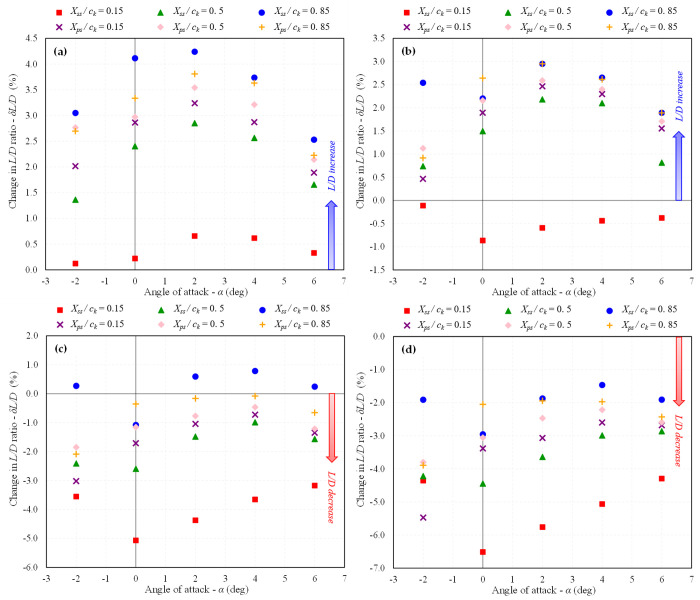
Variation in *L/D* ratio w.r.t baseline BWB at *Mach* 0.15; (a) *d/D*_*d*_ = 0.025; (b) 0.05; (c) 0.075; (d) 0.1.

The trend of increasing or decreasing *C*_*D*_ and *L/D* ratio in percentage change for each studied dimple depth at different locations is depicted in Figs 15 and 16, respectively. It is evident that the maximum drag reduction is achieved for *d* = 0.025*D*_*d*_ at nearly all of the studied dimple locations, resulting in an increase in the *L/D* ratio. As the dimple depth is increased, the efficacy of the dimples starts to decrease. The dimples still exhibit a trend of drag reduction for *d* = 0.05*D*_*d*_, but beyond this depth, drag begins to increase, and the *C*_*D*_ for *d* = 0.1*D*_*d*_ increases for all placement surfaces, leading to a decrease in the *L/D* ratio. This indicates that introducing deeper dimples does not provide any advantage in terms of the aerodynamic performance of dimples.

**Fig 17:**
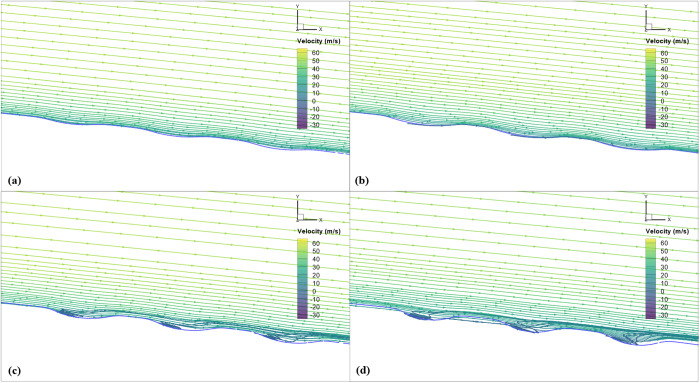
Velocity streamlines of all studied dimple depths for *X*_*ss*_*/c*_*k*_ = 0.85 at *Mach* 0.15, α = 2°; (a) *d/D*_*d*_ = 0.025; (b) 0.05; (c) 0.075; (d) 0.1.

The superiority of shallow dimples over deeper ones in terms of aerodynamic efficiency can be explained by considering the flow behavior and boundary layer interaction as illustrated through streamlines shown in Fig 17. Shallow dimples exhibit the best aerodynamic performance due to their ability to effectively control flow separation and minimize drag. Deeper dimples, on the other hand, tend to generate more turbulence within the boundary layer, leading to increased drag. Additionally, deeper dimples may cause flow separation, further exacerbating drag. Therefore, the diminishing efficacy observed with increasing dimple depth underscores the importance of maintaining shallow dimple profiles to optimize aerodynamic performance.

Considering the location, dimples placed near the TE on the suction surface are the most effective in terms of drag reduction and increased *L/D* ratio. The second-best location for their placement is observed to be near the TE on the pressure side. The least favorable location for dimple placement is observed near the LE on the suction surface, where the dimples cause an increase in drag for all studied indentation depths except for *d* = 0.025*D*_*d*_. The improved performance of dimples near the TE compared to those near the LE can be attributed to their ability to delay flow separation and minimize drag. Dimples placed near the TE effectively energize the boundary layer, delaying separation and promoting smoother airflow over the wing surface, resulting in reduced pressure drag and improved overall aerodynamic efficiency. In contrast, dimples near the LE may disrupt the flow in a less favorable manner, potentially causing premature separation and increased drag. Therefore, the location of dimples plays a crucial role in determining their aerodynamic effectiveness, with those near the TE exhibiting superior aerodynamic performance due to their ability to influence boundary layer behavior more effectively.

In summary, the maximum reduction in *C*_*D*_ of 4.09% at an AOA of 2° was obtained for the configuration, *d* = 0.025*D*_*d*_; *X*_*ss*_*/c*_*k*_ = 0.85, which led to an increase in *L/D* ratio of 4.24%. These findings suggest that shallow dimples with a depth of 2.5% of the diameter, particularly when placed near the TE on the suction surface, offer the most promising aerodynamic benefits in terms of drag reduction for the BWB airframe. These results provide valuable insights for optimizing the design of dimples on future BWB configurations, emphasizing the importance of considering both dimple depth and location for achieving optimal aerodynamic performance.

### 5.5. Effect of dimple position and indentation depth at high subsonic flow

Conducting simulations at a higher subsonic speed of *Mach* 0.6 expands our understanding of dimples’ aerodynamic behavior at elevated velocities. At *Mach* 0.6, the flow dynamics significantly differ from those observed at *Mach* 0.15 due to increased airspeed and corresponding alterations in flow patterns, boundary layer characteristics, and aerodynamic forces. Exploring the effects of dimples at this higher speed provides valuable insights into their performance in scenarios such as high-speed cruising and transonic flight regimes. This allows for a more comprehensive evaluation of their effectiveness across a broader range of flight conditions.

**Fig 18:**
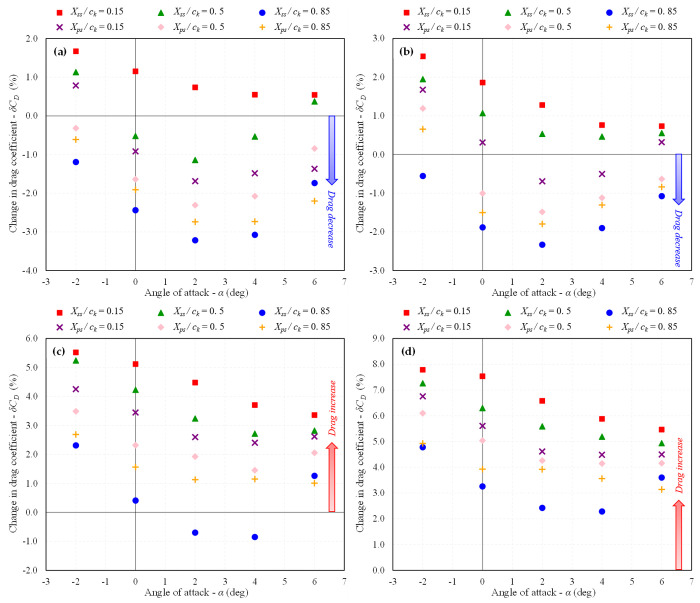
Variation in *C*_*D*_ w.r.t baseline BWB at *Mach* 0.6; (a) *d/D*_*d*_ = 0.025; (b) 0.05; (c) 0.075; (d) 0.1.

**Fig 19:**
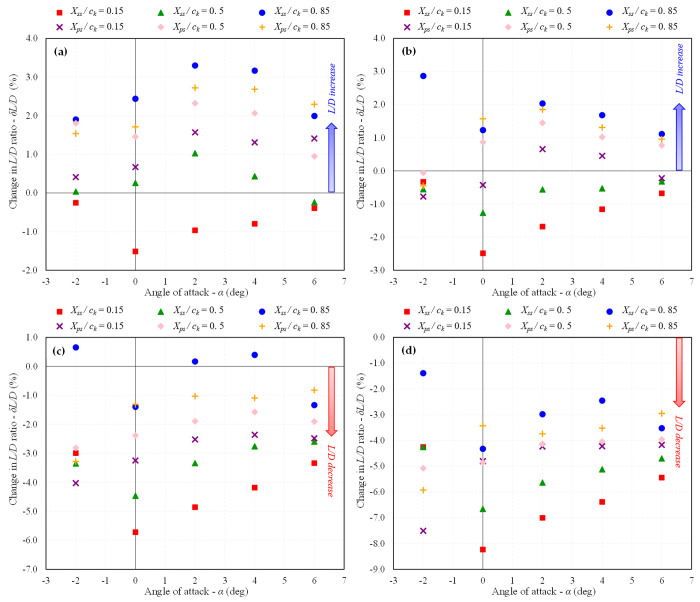
Variation in *L/D* ratio w.r.t baseline BWB at *Mach* 0.6; (a) *d/D*_*d*_ = 0.025; (b) 0.05; (c) 0.075; (d) 0.1.

**Fig 20:**
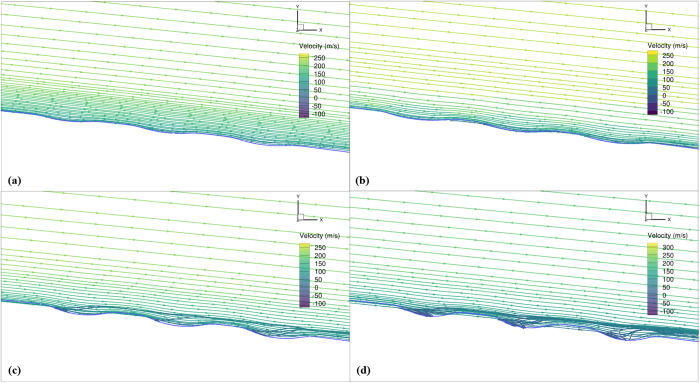
Velocity streamlines of all studied dimple depths for *X*_*ss*_*/c*_*k*_ = 0.85 at *Mach* 0.6, α = 2°; (a) *d/D*_*d*_ = 0.025; (b) 0.05; (c) 0.075; (d) 0.1.

The trends of increasing or decreasing *C*_*D*_ and *L/D* ratio in percentage change for each studied dimple depth at different locations at *Mach* 0.6 are shown in Figs 18 and 19, respectively and the velocity streamlines are shown in Fig 20. In terms of dimple indentation depth and location, similar trends are observed at *Mach* 0.6 as at *Mach* 0.15. Specifically, shallow dimples are found to be more effective compared to deeper ones, and dimples positioned near the TE on the suction surface exhibit superior aerodynamic performance compared to those near the LE. However, it is noteworthy that the efficacy of dimples in terms of drag reduction and *L/D* ratio improvement was observed to be reduced at *Mach* 0.6 compared to *Mach* 0.15.

The reduced effectiveness of dimples at high subsonic flow as compared to low subsonic flow can be attributed to the differences in airflow behavior and boundary layer characteristics between the two speed regimes. At low speeds, such as *Mach* 0.15, the flow around the aircraft is characterized by lower velocities, resulting in a thicker boundary layer. Dimples introduced under these conditions disrupt the boundary layer more effectively, leading to increased flow attachment and reduced separation, ultimately resulting in improved aerodynamic performance in terms of drag reduction. In contrast, at higher speeds like *Mach* 0.6, the flow velocity is higher, and the boundary layer is thinner. This makes it more challenging for the dimples to effectively disrupt the flow, leading to reduced aerodynamic benefits compared to low-speed conditions.

In summary, at *Mach* 0.6, the maximum reduction in *C*_*D*_ of 3.22% at an AOA of 2° was obtained for the configuration, *d* = 0.025*D*_*d*_; *X*_*ss*_*/c*_*k*_ = 0.85, which led to an increase in *L/D* ratio of 3.30%. In summary, with careful design and optimization, integrating dimples on lifting surfaces presents a promising approach for improving aerodynamic performance. Moreover, conducting simulations at low and high subsonic velocities has enabled us to understand how dimples affect the aerodynamic phenomena associated with the BWB across different operational speeds. This approach has enhanced the robustness of our analysis and facilitated a more thorough assessment of the efficacy of dimples in improving the overall aerodynamic performance of the BWB airframe.

### 5.6. Quantitative trend analysis

To quantitatively assess how dimple depth affects aerodynamic performance, a regression analysis was conducted based on the percentage change in aerodynamic parameters at the optimal dimple location *X*_*ss*_*/c*_*k*_ = 0.85 and AOA = 2°. This specific AOA was selected as it consistently yielded the maximum drag reduction and *L/D* ratio enhancement across both *Mach* numbers in the parametric simulations. The *d/D*_*d*_ was varied across all studied levels.

A second-order polynomial regression was fitted to quantify the relationship between indentation depth and drag reduction. The equations derived from the curve fits are as follows:


$${\left(\delta C_{D}\right)}_{Mach\;0.15}=\;589.20{\left(\frac{d}{D_{d}}\right)}^{2}+2.50\left(\frac{d}{D_{d}}\right)-4.55$$ (16)

$${\left(\delta C_{D}\right)}_{Mach\;0.6}=\;896{\left(\frac{d}{D_{d}}\right)}^{2}-37.82\left(\frac{d}{D_{d}}\right)-2.79$$ (17)

**Fig 21:**
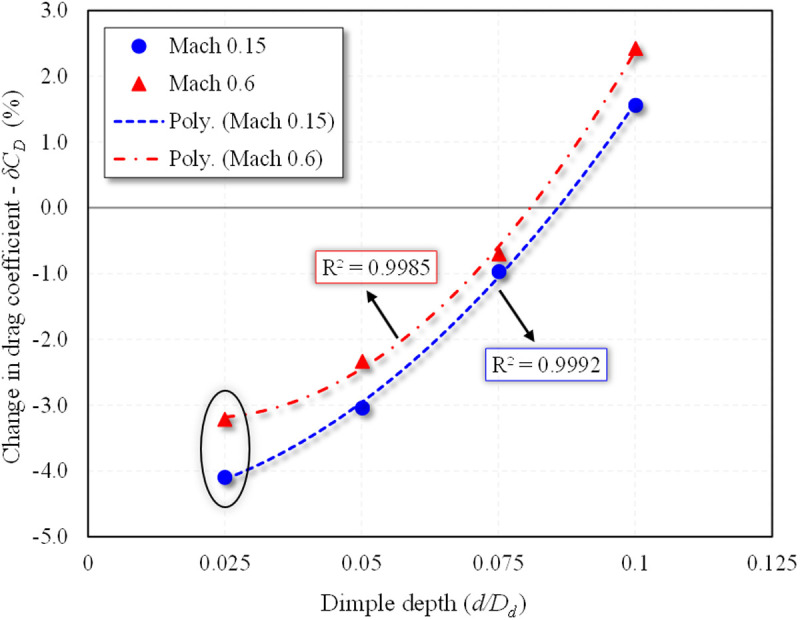
Polynomial regression analysis of δ*C*_*D*_ (%) w.r.t to dimple depths for *X*_*ss*_*/c*_*k*_ = 0.85 at α = 2°.

These trend lines, shown in Fig 21, reveal a clearly defined optimum at *d/D*_*d*_ = 0.025 which corresponds to the maximum drag reduction. As the indentation depth increases beyond this point, the aerodynamic performance deteriorates, with drag reduction decreasing significantly and eventually becoming positive (i.e., resulting in drag penalties). This outcome aligns with physical flow behavior, deeper dimples generate more intense localized recirculation zones, which in turn thicken the boundary layer and lead to increased pressure drag.

Further, the impact of dimple depth on the percentage change in *L/D* ratio was evaluated. The regression equations are:


$${\left(\delta L/D\right)}_{Mach\;0.15}=\;-468.52{\left(\frac{d}{D_{d}}\right)}^{2}-24.207\left(\frac{d}{D_{d}}\right)+5.185$$ (18)

$${\left(\delta L/D\right)}_{Mach\;0.6}=\;-752.37{\left(\frac{d}{D_{d}}\right)}^{2}+11.208\left(\frac{d}{D_{d}}\right)+3.456$$ (19)

**Fig 22:**
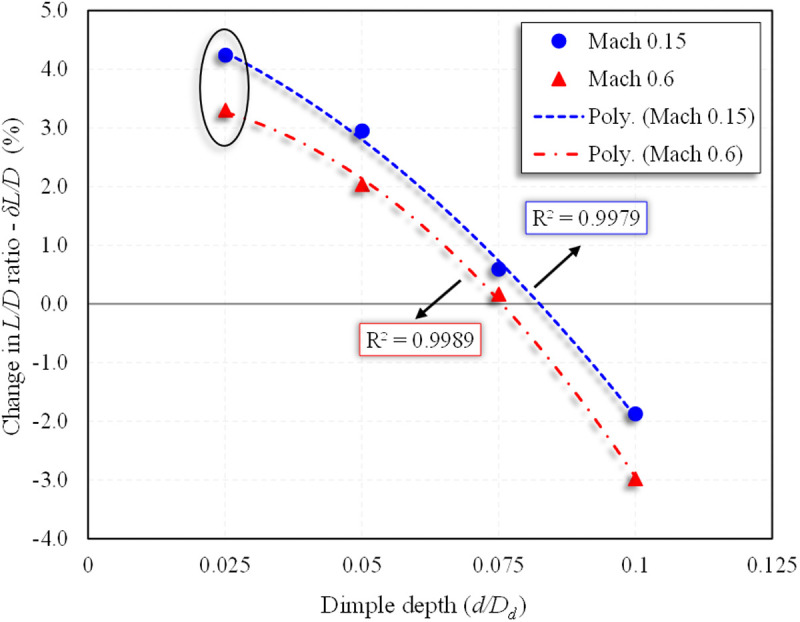
Polynomial regression analysis of δ*L/D* (%) w.r.t to dimple depths for *X*_*ss*_*/c*_*k*_ = 0.85 at α = 2°.

These relationships shown in Fig 22 confirm that maximum aerodynamic efficiency, as measured by the percentage improvement in *L/D* ratio, also occurs at *d/D*_*d*_ = 0.025. These findings substantiate that small dimple depths promote attached flow while minimizing form drag, enhancing the aircraft’s net aerodynamic efficiency. Importantly, the high coefficients of determination (*R*^*2*^ > 0.99) confirm the robustness of the regression models and validate the reliability of the CFD-based results.

Considering that deeper dimples lead to performance degradation, while shallow dimples were most effective in enhancing aerodynamic performance, further exploration and optimization of dimple design parameters are crucial for improved flow control. Although exploring smaller indentation depths below 2.5% might be interesting, the RANS solver used in this study may not effectively capture the subtle effects of such small surface modifications. While high-fidelity simulations such as LES or DNS could offer more detailed insights into the effects of very shallow dimples, these methods are computationally intensive and were not feasible within the scope of this study. Future work could employ these approaches to refine the understanding of dimple effects, particularly in optimization studies that systematically explore finer variations in dimple indentation depth. Moreover, variables such as dimple diameter, curvature radius, streamwise and spanwise spacing, and alternative dimple shapes beyond spherical also present promising avenues for exploration in subsequent research.

While our study suggests that incorporating dimples on BWB configurations can improve aerodynamic characteristics, it’s crucial to explore their viability from a manufacturing standpoint. Firstly, it’s essential to evaluate the feasibility and cost-effectiveness of integrating dimples into the manufacturing process of the BWB or any other modern aircraft. Furthermore, the impact of dimples on the structural integrity and durability of the BWB airframe must be thoroughly assessed. Structural analyses, such as finite element modeling, can help determine how dimples affect stress distribution and fatigue life, ensuring that the structural integrity of the aircraft is maintained over its operational lifespan.

## 6. Conclusion

This study employs CFD analysis to comprehensively investigate the aerodynamic impact of dimples introduced onto the wing surfaces of a BWB airframe. The parametric investigation examines the influence of four distinct dimple indentation depths at six specific locations on either the suction or pressure sides of the BWB wings. A total of 24 dimpled BWB configurations are investigated at *Mach* 0.15 and *Mach* 0.6, across an AOA spanning from −2° to 6°, providing a thorough insight of the impact of dimples on the aerodynamic behaviour of the BWB under varying flow conditions and different flight regimes. The resolution of governing continuity and momentum equations is accomplished through a RANS solver coupled with the *k – ω* (SST) turbulent model.

The results suggest that, with appropriate design, dimples have the potential to substantially enhance the aerodynamic properties of lifting surfaces. Notably, shallow dimples demonstrated superior aerodynamic performance compared to deeper ones. The study observed that increasing the depth of dimples led to decreased efficacy, and deeper dimples even resulted in increased drag, underscoring the importance of optimal dimple design. Furthermore, the analysis revealed that dimples positioned near the TE on the suction surface exhibited the most significant drag reduction. Similar trends are observed at both *Mach* numbers in terms of dimple indentation depth and location, although the aerodynamic efficacy of dimples is reduced at *Mach* 0.6 compared to *Mach* 0.15. Among the various BWB configurations studied, the configuration with *d* = 0.025*D*_*d*_ at *X*_*ss*_*/c*_*k*_ = 0.85 exhibited the best performance. This configuration yields a maximum reduction in *C*_*D*_ of 4.09% at an AOA of 2° at *Mach* 0.15. Notably, this reduction does not adversely impact the *C*_*L*_, resulted in an improved *L/D* ratio of 4.24%. At *Mach* 0.6, the same configuration achieved a maximum reduction in *C*_*D*_ of 3.22% with an improved *L/D* ratio of 3.3%.

To deepen the physical understanding of these improvements, *Cp* distributions were analyzed, illustrating how dimples manipulate local pressure gradients and stabilize the boundary layer. Moreover, regression-based mathematical models were developed to quantify the relationship between dimple depth and aerodynamic response, revealing a distinct parabolic trend with optimal performance at a shallow depth of *d* = 0.025*D*_*d*_.

Our study emphasizes the potential impact of strategically positioned dimples on lifting surfaces, offering a passive means to manipulate airflow and consequently enhance aerodynamic performance. This advancement is particularly significant given the aviation industry’s current focus on cleaner and more fuel-efficient solutions, driven by increasing air traffic and environmental concerns. However, it’s crucial to recognize that our numerical simulations are based on specific assumptions and simplifications. Future recommendations include validating these findings through experimental wind tunnel tests. Such real-world experimentation holds the key to fully realizing the benefits of dimples in shaping the future of aerodynamics and driving innovation within the aviation sector.

In summary, the ability of dimples to maintain attached airflow and delay flow separation holds significant potential for improving the overall efficiency, maneuverability, and safety of BWB aircraft design. Further research and experimentation in real-world flight conditions will continue to refine and validate the effectiveness of this innovative aerodynamic solution. Overall, this study, alongside similar research, advances the pursuit of sustainable and eco-friendly aviation. It represents a crucial step toward developing universally optimized aerodynamic configurations that can benefit the aviation industry under a wide array of operational conditions.
